# Tuberculous pleural effusion-induced Arg-1^+^ macrophage polarization contributes to lung cancer progression via autophagy signaling

**DOI:** 10.1186/s12931-024-02829-8

**Published:** 2024-05-08

**Authors:** Seong Ji Woo, Youngmi Kim, Hyun-Jung Kang, Harry Jung, Dong Hyuk Youn, Yoonki Hong, Jae Jun Lee, Ji Young Hong

**Affiliations:** 1 Institute of New Frontier Research Team, Hallym University College of Medicine, Chuncheon, Republic of Korea; 2 Department of Internal Medicine, School of Medicine, Kangwon National University, Kangwon National University Hospital, Chuncheon, Republic of Korea; 3 Division of Pulmonary and Critical Care Medicine, Department of Medicine, Chuncheon Sacred Heart Hospital, Hallym University Medical Center, Chuncheon, Republic of Korea; 4 Department of Internal Medicine, Hallym University Chuncheon Hospital, Chuncheon, South Korea

**Keywords:** Tuberculosis, Lung cancer, Macrophage, Arginase-1, Treatment

## Abstract

**Background:**

The association between tuberculous fibrosis and lung cancer development has been reported by some epidemiological and experimental studies; however, its underlying mechanisms remain unclear, and the role of macrophage (MФ) polarization in cancer progression is unknown. The aim of the present study was to investigate the role of M2 Arg-1^+^ MФ in tuberculous pleurisy-assisted tumorigenicity in vitro and in vivo.

**Methods:**

The interactions between tuberculous pleural effusion (TPE)-induced M2 Arg-1^+^ MФ and A549 lung cancer cells were evaluated. A murine model injected with cancer cells 2 weeks after *Mycobacterium bovis* bacillus Calmette–Guérin pleural infection was used to validate the involvement of tuberculous fibrosis to tumor invasion.

**Results:**

Increased CXCL9 and CXCL10 levels of TPE induced M2 Arg-1^+^ MФ polarization of murine bone marrow-derived MФ. TPE-induced M2 Arg-1^+^ MФ polarization facilitated lung cancer proliferation via autophagy signaling and E-cadherin signaling in vitro. An inhibitor of arginase-1 targeting M2 Arg-1^+^ MФ both in vitro and in vivo significantly reduced tuberculous fibrosis-induced metastatic potential of lung cancer and decreased autophagy signaling and E-cadherin expression.

**Conclusion:**

Tuberculous pleural fibrosis induces M2 Arg-1^+^ polarization, and M2 Arg-1^+^ MФ contribute to lung cancer metastasis via autophagy and E-cadherin signaling. Therefore, M2 Arg-1^+^ tumor associated MФ may be a novel therapeutic target for tuberculous fibrosis-induced lung cancer progression.

**Supplementary Information:**

The online version contains supplementary material available at 10.1186/s12931-024-02829-8.

## Background

Although several epidemiological studies have reported an association between tuberculosis (TB) and cancer development, the underlying mechanisms remain unclear [1–3].

The metabolic function and immune properties of macrophages (MФ) in response to *M. tuberculosis* infection are associated with pathogenicity and outcomes [4]. In the late adaptation/resolution phase, a metabolic shift occurs, and increased PGC-1 beta level promotes M2 polarization and inhibits proinflammatory and antimicrobial responses [5]. M2 MФ predominate in granulomatous lung tissues compared to M1 MФ [6]. Exosomal miRNA from M2 MФ promotes the progression of pulmonary fibrosis [7].

Tumor-associated MФ (TAM) play a pro-tumoral role in the tumor microenvironment by enhancing the rate of tumor cell invasion, extravasation, and growth [8]. The immunosuppressive TAM (M2) may inhibit natural killer cells and T cells during tumor progression [9].

In a previous study, we found that the NOX4/autophagy axis mediates tuberculous fibrosis-induced tumorigenicity [10]. Helfinger et al. found that NOX4 regulates MФ polarization [11]. Zhang et al. reported that tumoral NOX4-educating M2 MФ promote lung cancer growth [12]. Based on these results, we hypothesized that MФ polarization after TB infection contributes to a microenvironment that is susceptible to tumor progression and acts as a new therapeutic target.

In this study, we aimed to examine the effect of tuberculous pleural effusion (TPE)-induced Arg-1 M2 MФ polarization on the invasion of A549 cells using an in vitro co-culture method. We evaluated the treatment efficacy of inhibiting Arg-1 M2 MФ polarization in TB-associated lung cancer in vitro and in vivo using a *Mycobacterium bovis* bacillus Calmette–Guérin (BCG)-induced pleurisy mouse model.

## Methods

### Cell lines and animals

The human adenocarcinoma cell line A549, mouse musculus lung squamous cell line KLN205, and mouse fibroblast cell line L-929 (American Type Culture Collection, Manassas, VA, USA) were cultured according to the manufacturer’s instructions. A549 cells were maintained in RPMI 1640 medium (BYLABS, Hanam, Korea) supplemented with 10% FBS, 100 U/mL penicillin, and 100 μg/mL streptomycin at 37 °C in a humidified incubator with 5% CO_2_. KLN205 cells were maintained in Eagle’s Minimum Essential medium (Corning, Manassas, VA, USA) supplemented with 10% FBS, 100 U/mL penicillin, and 100 μg/mL streptomycin at 37 °C in a humidified incubator with 5% CO_2_. L-929 cells were maintained in DMEM/F12 (Thermo Fisher Scientific, Waltham, MA, USA) supplemented with 10% horse serum at 37 °C in a humidified incubator with 5% CO_2_. L-929 cell culture medium (conditioned medium, L-929 culture medium (CM)) was collected and centrifuged at 3,000 rpm and 4 °C, passed through a 0.45-µm filter, and frozen at -27 °C in 50 mL aliquots.

Wild-type C57BL/6J mice (DooYeol Biotech, Seoul, Korea) were bred in a vivarium at Hallym University (Chuncheon, Korea). All animal experiments were approved by the Institutional Animal Care and Use Committee of Hallym University (No: Hallym 2017-47).

### Reagents and kits

Human XL Cytokine Array Kit (R&D Systems, Minneapolis, MN, USA) was used to compare cytokine profiles between TPE and transudate (T). Recombinant CXCL9 and CXCL10 and neutralizing antibodies against CXCL9 and CXCL10 were purchased from R&D Systems. The arginase inhibitor 2(S)-amino-6-boronohexanoic acid (ABH) was purchased from Cayman Chemicals (Ann Arbor, Michigan, USA) and used in cell and mouse experiments. The autophagy inhibitors 3-methyladenine (3-MA) and bafilomycin A1 (BafA1) were purchased from Enzo Life Sciences (Farmingdale, NY, USA).

### Bone marrow-derived MФ differentiation and polarization

**Fig. 1:**
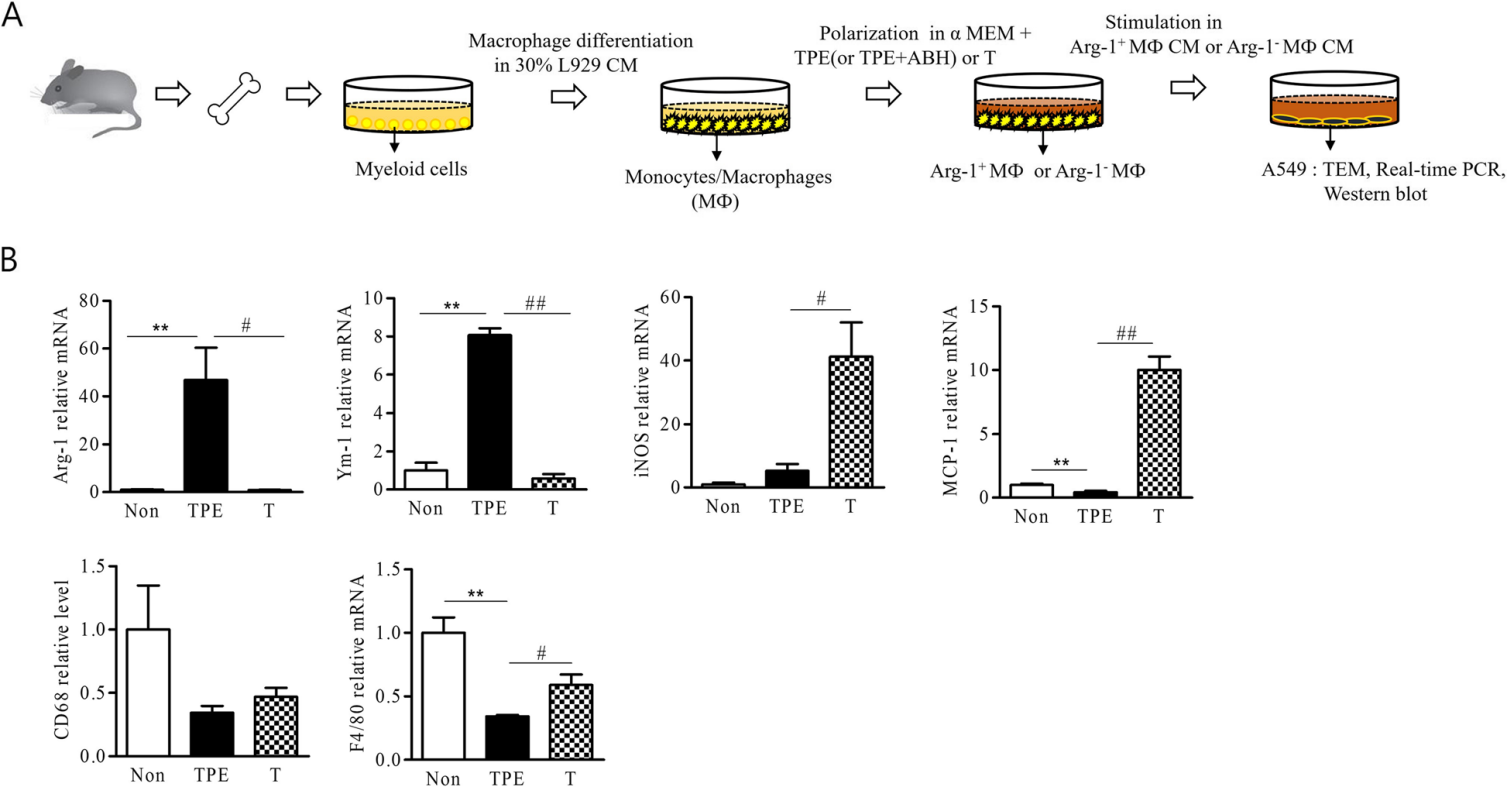
Tuberculous pleural effusion (TPE) leads to increased M2 polarization of macrophages. **a** The schematic diagram shows the production of TPE-Arg-1^+^ MФ CM. BMDMs were treated with TPE or transudate (T) for 48 h. After MФ polarization, TPE-Arg-1^+^ MФ CM or T-Arg-1^−^ MФ CM was added to A549 cells and incubated for 48 h. **b** The M2 (Arg-1 and YM-1), M1 (iNOS and MCP-1), and pan-macrophage markers (CD68 and F4/80) markers were quantified using RT-qPCR after stimulation with TPE or T. The values represent the results of three experiments. **p < 0.01, #*p* < 0.05, ##*p* < 0.01

MФ were derived from the bone marrow cells of 6–7-week-old C57BL/6J mice. The cells were differentiated for 6 days in α-minimum essential medium supplemented with 30% L-929 CM. The bone marrow-derived MФ (BMDMs) were treated with 1:1 dilution of TPE or T for 48 h to induce polarization. Thereafter, the MФ supernatant (TPE-Arg-1^+^ MФ CM or T-Arg-1- MФ CM) was harvested and used to treat A549 cells (Fig. 1a).

### RNA extraction, real-time polymerase chain reaction, western blotting, and enzyme-linked immunosorbent assay

Total RNA was extracted from A549 cells or mouse lung tissue samples using easy-Blue reagent (iNtRON Biotechnology, Seoul, Korea). First-strand cDNA was synthesized using the Maxime RT PreMix Kit (iNtRON Biotechnology) according to the manufacturer’s protocol. Real-time polymerase chain reaction (PCR) was performed on Rotor-Gene Q using SYBR Green I as a double-stranded DNA-specific dye according to the manufacturer’s instructions. The PCR mixture of total volume 20 μL comprised 1 μL cDNA, 10 μL *Power* SYBR Green PCR Master Mix (Thermo Fisher Scientific), and 10 pM each of forward and reverse primers. Relative expression was calculated as ddCt, and the data for each sample were normalized to the expression of *β-actin* mRNA. The sequences of primers used for each gene are presented in Table S1. For immunoblot analysis, the cells and tissues were lysed with RIPA buffer containing a protease inhibitor cocktail (GenDEPOT, Baker, TX, USA). Membranes were incubated overnight at 4 °C with the primary antibodies against actin, LC3B, ATG12, p-Beclin, Beclin, E-cadherin (Cell Signaling Technology, Danvers, MA, USA), ZO-1 (Abcam, Cambridge, UK), p62 (Santa Cruz Biotechnology, Dallas, TX, USA), and Bcl-2 (Enzo Life Sciences). After washing the blots in TBS containing Tween-20, they were incubated with horseradish peroxidase-conjugated secondary antibodies corresponding to each primary antibody. Signals were detected using the ECL detection kit (Thermo Fisher Scientific). Mouse TGF-β or Arginase-1 in serum samples was measured using an ELISA kit (Abbkine, Inc., Atlanta, Georgia, USA), according to the manufacturer’s protocol.

### Cell invasion assay

A549 cell or MФ invasion assay was performed using Matrigel Transwell chambers with a pore size of 8 μm (Corning). For A549 cell invasion assay, A549 cells were seeded in the upper chamber and the CM of MФ (TPE-Arg-1^+^ + MФ, T-Arg-1^−^ + MФ, and ABH + TPE Arg-1^+^ + MФ) was added to the lower chamber. For the MФ invasion assay, TPE-Arg-1^+^ + MФ or T-Arg-1^−^ + MФ were seeded (1 × 10^5^ cells/well) in the upper chamber and A549 cells were seeded in the lower chamber. After 24 h, the invasive cells that penetrated the Matrigel barrier to the lower surface were stained with crystal violet solution and counted.

### Transmission electron microscopy

A549 cells were treated with Arg-1^+^ MФ CM or Arg-1^−^ MФ CM and ABH + TPE Arg-1^+^ CM for 48 h and examined using a transmission electron microscope (FE-TEM, JEM 2100F; Jeol, Japan). A modified Karnovsky’s fixative was used to identify autophagosomes.

### Animal experiments

To develop a BCG-induced pleural fibrosis model, mice were injected with 10^6^ colony forming units of BCG Pasteur in 100 µL of PBS into the intrapleural cavity; after 2 weeks, mouse musculus lung squamous cells (KLN205; ATCC) were intravenously injected. ABH (200 μg/mouse) was administered intraperitoneally every other day for 22 days from the day of BCG injection. On day 10 after KLN205 injection, the mice were anesthetized with 4% isoflurane (Piramal Critical Care, Bethlehem, PA, USA), and then lung tissue and serum samples were collected. Paraffin-embedded lung tissue samples were sectioned to 5-μm thickness and stained with hematoxylin and eosin. Histological changes were imaged under a light microscopy (ZEISS Colibri 7, Land Baden-Württemberg, Germany). The lung tissue sections were immunostained with antibodies against collagen (Invitrogen), Arginase-1 (LSBio, Shirley, MA, USA), LC3B (Cell Signaling Technology, Danvers, MA, USA), and F4/80 (Abcam, Cambridge, UK). The samples were analyzed under a fluorescence microscope (Olympus FV500; Olympus, Tokyo, Japan). DAPI (Sigma, St. Louis, MO, USA) was used as a counterstain.

### Human samples

The study was approved by the hospital’s research ethics committee (institutional review board numbers: 2012-27 and 2017-47). Written informed consent was obtained from all participants before sample collection. Pleural effusion samples were obtained via routine thoracentesis. The definitions of TPE and T were consistent with those of a previous study [13]. Pleural effusion samples were collected in sterile tubes and centrifuged at 3,000 g for 10 min and the cell-free supernatant was stored at −80 °C for analysis.

### Statistical analyses

All experiments were conducted in triplicate. GraphPad Prism (V.7, GraphPad, USA) was used for statistical analyses. Quantification of lung metastasis lesions were performed using Qupath software (https://qupath.github.io/). Variables are presented as mean ± standard error of the mean. An unpaired Student’s *t*-test was used to compare the differences between means. Statistical significance was set at *p* < 0.05.

## Results

### TPE induces M2 Arg-1^+^ MФ polarization

Figure 1a shows a schematic representation of the experimental procedure. To evaluate whether TPE-induced Arg-1 + MФ polarization promotes A549 cell growth in vitro, we first treated differentiated MФ with TPE or T for 48 h, and then, MФ CM (Arg-1^+^ MФ CM or Arg-1^−^ MФ CM) was added to A549 cells and incubated for 48 h to determine the growth of A549 cells. Subsequently, the A549 cells were analyzed using TEM, real-time PCR, and western blotting. Compared to T, TPE treatment upregulated the expression of M2-associated makers Arg-1 and Ym-1, but did not increase that of the M1-associated markers MCP-1 and iNOS. TPE treatment did not induce cell death (Fig.S1).

### TPE-Arg-1^+^ promotes A549 cell proliferation through autophagy signaling

**Fig. 2:**
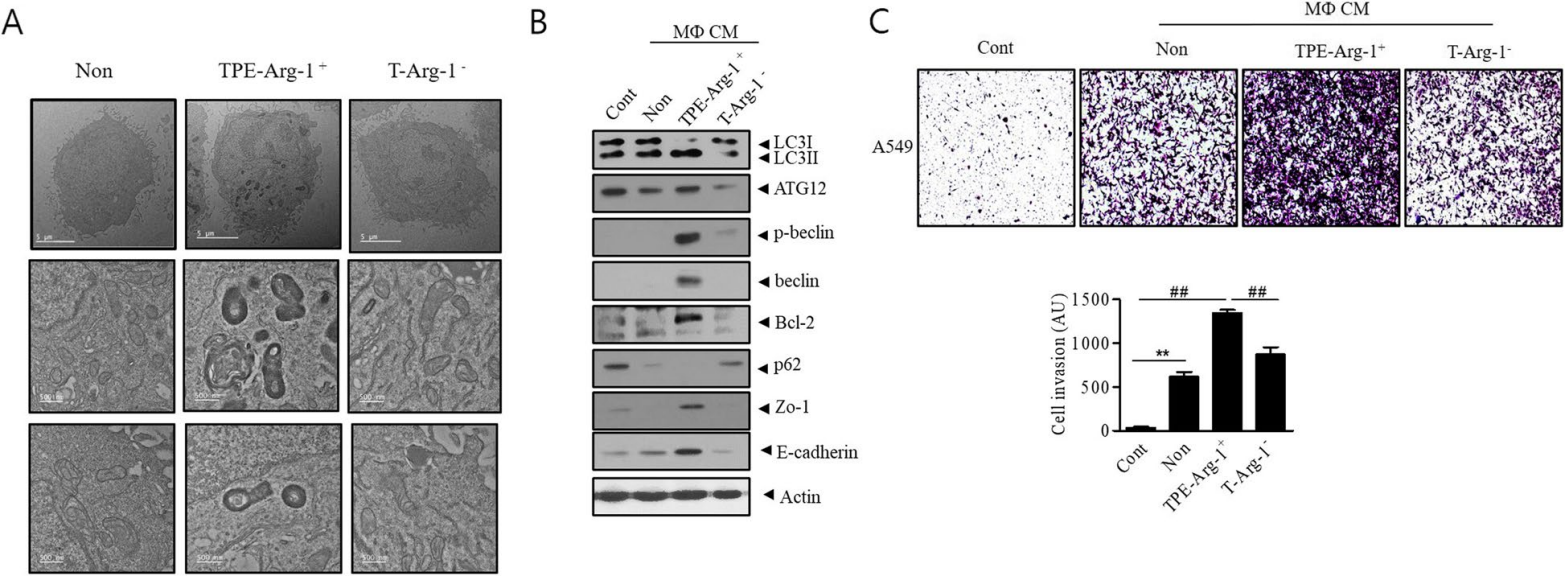
TPE-educated Arg-1^+^ macrophages promote A549 cell growth in vitro by upregulating autophagy and E-cadherin signaling. **a** Transmission electron microscopy showed that TPE-Arg-1^+^ MФ CM increased autophagic vesicle abundance in A549 cells compared to T-Arg-1^−^ MФ CM (Scale bar: upper row: 5 μm, middle and lower row: 500 nm). **b** Western blotting of autophagy-related proteins (LC3II /LC3I, ATG12, p-Beclin, Beclin, BCL-2, and P62), ZO-1 and E-cadherin in A549 cells treated with TPE-Arg-1^+^ MФ or, T-Arg-1^−^ MФ CM. **c** Transwell assay showed that TPE-Arg-1^+^ MФ significantly increased the invasion of lung adenocarcinoma cells compared to T-Arg-1^−^ MФ CM. Data represent the results of three experiments.**p < 0.01, ##*p* < 0.01

Electron microscopy showed that TPE-Arg-1^+^ MФ CM-treated A549 cells had numerous autophagosomes. The number of autophagosomes in the TPE-Arg-1^+^ MФ CM group was significantly higher than that in the control and T- Arg-1^−^ MФ CM groups (Fig. 2a). TPE-Arg-1^+^ MФ CM increased the expression of LC3, ATG12, Beclin, and Bcl-2, but decreased that of P62 in A549 cells (Fig. 2b). The CM of the TPE-Arg-1^+^ MФ CM group increased the expression of E-cadherin and ZO-1 (Fig. 2b). TPE-Arg-1^+^ MФ CM enhanced the invasion and migration of A549 cells compared to the control and T- Arg-1- MФ CM (Fig. 2c). This result suggests that TPE-Arg-1^+^ MФ CM activates autophagy and E-cadherin signaling as cancer progression pathways. Autophagosome accumulation appears to be induced by autophagosome formation rather than autophagic flux. Exposing the TPE-Arg-1^+^ MФ CM-treated A549 cells to BafA1 did not change the conversion of LC3-II from LC3-I but 3-MA treatment reduced ATG12 and LC3-II levels compared with those in the TPE-Arg-1^+^ MФ CM-treated A549 cells (Fig.S2).

To determine the effect of A549 cells on MФ migration, murine MФ were seeded in the upper chambers of Transwell chambers and treated with the CM of A549 cells for 48 h (Fig.S3). The migration rate of TPE-Arg-1^+^ MФ increased compared with that of the control and T- Arg-1^−^ MФ groups. These results indicate that the influence of cancer cells on MФ migration increased during TPE treatment, and consequently, the interaction between the cell types was amplified.

### CXCL9 and CXCL10 in TPE mediate Arg-1^+^ MФ polarization

**Fig. 3:**
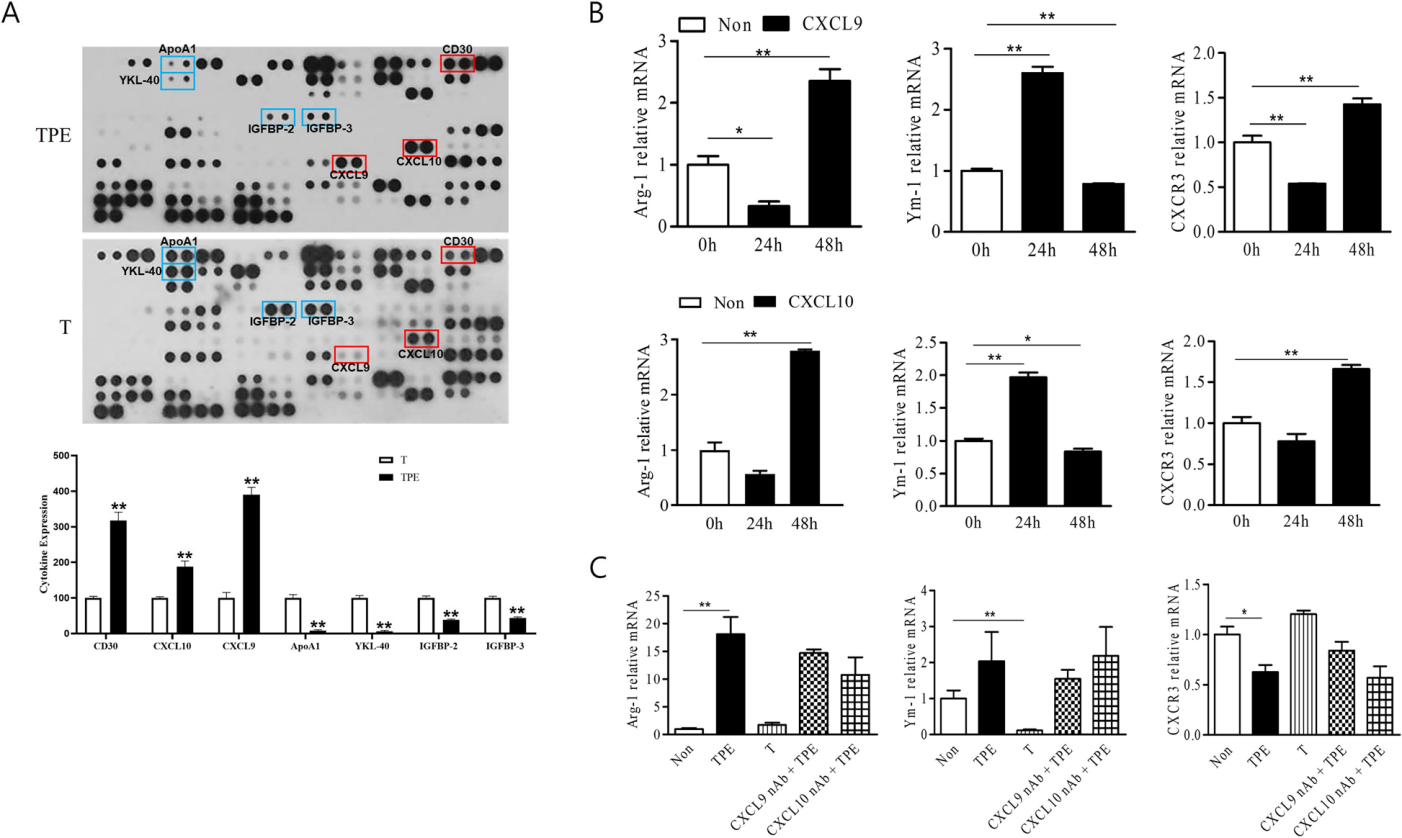
CXL9 and CXL10 in TPE modulate M2 macrophage polarization. **a** Cytokine array analysis demonstrated differences between TPE and T. Spots with differentially regulated cytokines are identified with a red box and a blue box. Red boxes correspond to CD30, CXCL9, and CXCL10. Blue boxes correspond to ApoA1, YKL-40, IGFBP-2, and IGFBP-3. **b** Mouse BMDMs were treated with 20 µg recombinant CXCL9 and 20 µg recombinant CXCL10. **c** Mouse BMDMs were cultured with neutralizing antibodies for CXCL9 (20 µg) and CXCL10 (20 µg) after TPE treatment. Arg-1 mRNA, Ym-1 mRNA, and CXCR3 mRNA levels were measured. ApoA1: apolipoprotein A1; YKL-40: chitinase-3-like protein 1; IGFBP-2: insulin like growth factor binding protein 2; IGFBP-3: insulin like growth factor binding protein 3. The values represent the results of three experiments. **p < 0.01, *p < 0.05

To investigate the TPE components responsible for MФ polarization, we performed a cytokine array analysis of TPE. CXCL9 and CXCL10 expression was upregulated in TPE compared with that in T, whereas apolipoprotein A-1, YKL-40, IGFBP-2, and IGFBP-3 expression was upregulated in T compared with that in TPE (Fig. 3a). To further confirm that CXCL9 and CXCXL10 regulate MФ M2 polarization, we performed chemokine stimulation and neutralization experiments on MФ. Stimulation with 20 µg CXCL9 and 20 µg CXCL10 increased Arg-1^+^ MФ polarization after 48 h (Fig. 3b). The level of CXCR3, the receptor of CXCL9 and CXCL10, decreased after 24 h, and then replenished at 48 h.

When TPE treatment of MФ was followed by the addition of CXCL9 antibody (4 µg) or CXCL10 antibody (4 µg) for 48 h, the Arg-1^+^ M2 MФ polarization rate decreased compared with that after only TPE treatment. These results suggest that CXCL9 and CXCL10 elicited by TB development may be involved in M2 MФ polarization.

### Arginase inhibition reduces the growth of A549 cells exposed to TPE by suppressing autophagy signaling

**Fig. 4:**
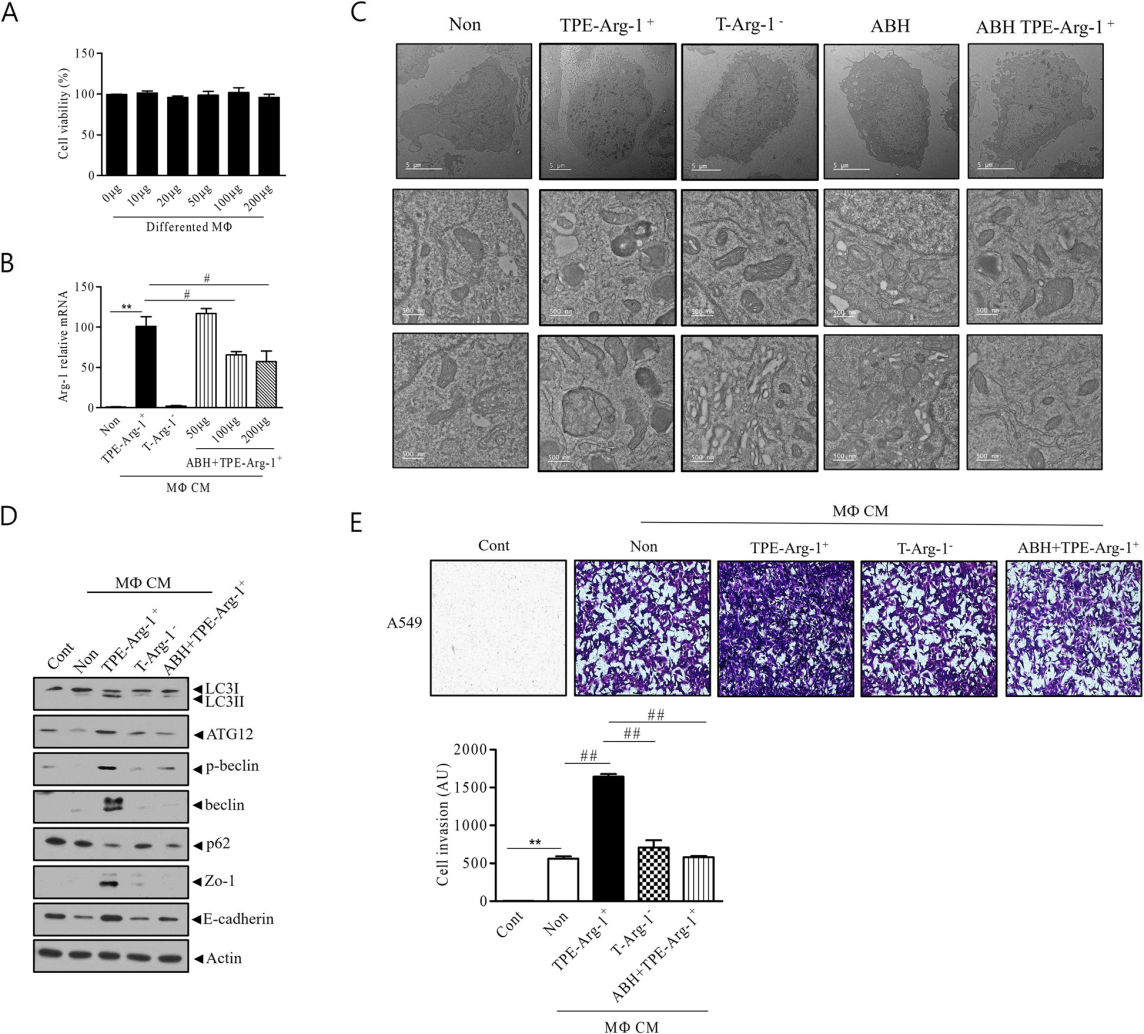
Arg-1 inhibitor reduces A549 cell growth in vitro. **a** Macrophage cell viability was measured following treatment with the Arg-1 inhibitor at different concentrations. **b** The Arg-1 mRNA level in A549 cells treated with TPE-Arg-1^+^ MФ CM, T-Arg-1^−^ MФ CM and CM of TPE-Arg-1^+^ MФ following pretreatment with the Arg-1 inhibitor (ABH + TPE-Arg-1^+^ MФ CM) was measured. **c** The autophagic vesicle abundance was lower in the ABH + TPE-Arg-1^+^ MФ CM group than in the TPE-Arg-1^+^ MФ CM group (Scale bar: upper row: 5 μm, middle and lower row: 500 nm). **d** The expression of autophagy-related proteins (P62, ATG12, LC3II /LC3I, p-Beclin, and Beclin), ZO-1, and E-cadherin decreased in A549 cells cultured with ABH + TPE-Arg-1^+^ MФ CM compared with that in cells cultured with TPE-Arg-1^+^ MФ CM. **e** The Transwell assay showed that ABH + TPE-Arg-1^+^ MФ significantly attenuated the invasion of lung adenocarcinoma compared to TPE-Arg-1^+^ MФ. The values represent the results of three experiments. **p < 0.01, ##*p* < 0.01, #*p* < 0.05

We performed experiments to select the concentrations of ABH at which cell viability is maintained and Arg-1 MФ polarization is reduced (Fig. 4a and b). BMDMs were incubated with 100 µg ABH for 24 h, and then with TPE for 48 h. The CM of ABH + TPE-Arg-1^+^ MФ was used to treat A549 cells for comparison with that of TPE-Arg-1^+^ MФ. Electron microscopy showed that the number of autophagosomes in the ABH + TPE-Arg-1^+^ CM group was significantly lower than that in the TPE-Arg-1^+^ MФ CM group, as confirmed using the Transwell invasion assay and western blotting (Fig. 4c).

The ABH + TPE-Arg-1^+^ CM group showed reduced expression of LC3, ATG12, Beclin, and Bcl-2, but increased expression of P62, compared to the TPE-Arg-1^+^ MФ CM group (Fig. 4d). In addition, the expression of E-cadherin and ZO-1 was lower in the ABH + TPE-Arg-1^+^ CM group than in the TPE-Arg-1^+^ MФ CM group (Fig. 4d). This result shows that ABH may downregulate autophagy and E-cadherin signaling induced by TPE-Arg-1^+^ MФ as cancer progression pathways.

### Effect of ABH in the BCG-induced lung cancer model in vivo

**Fig. 5:**
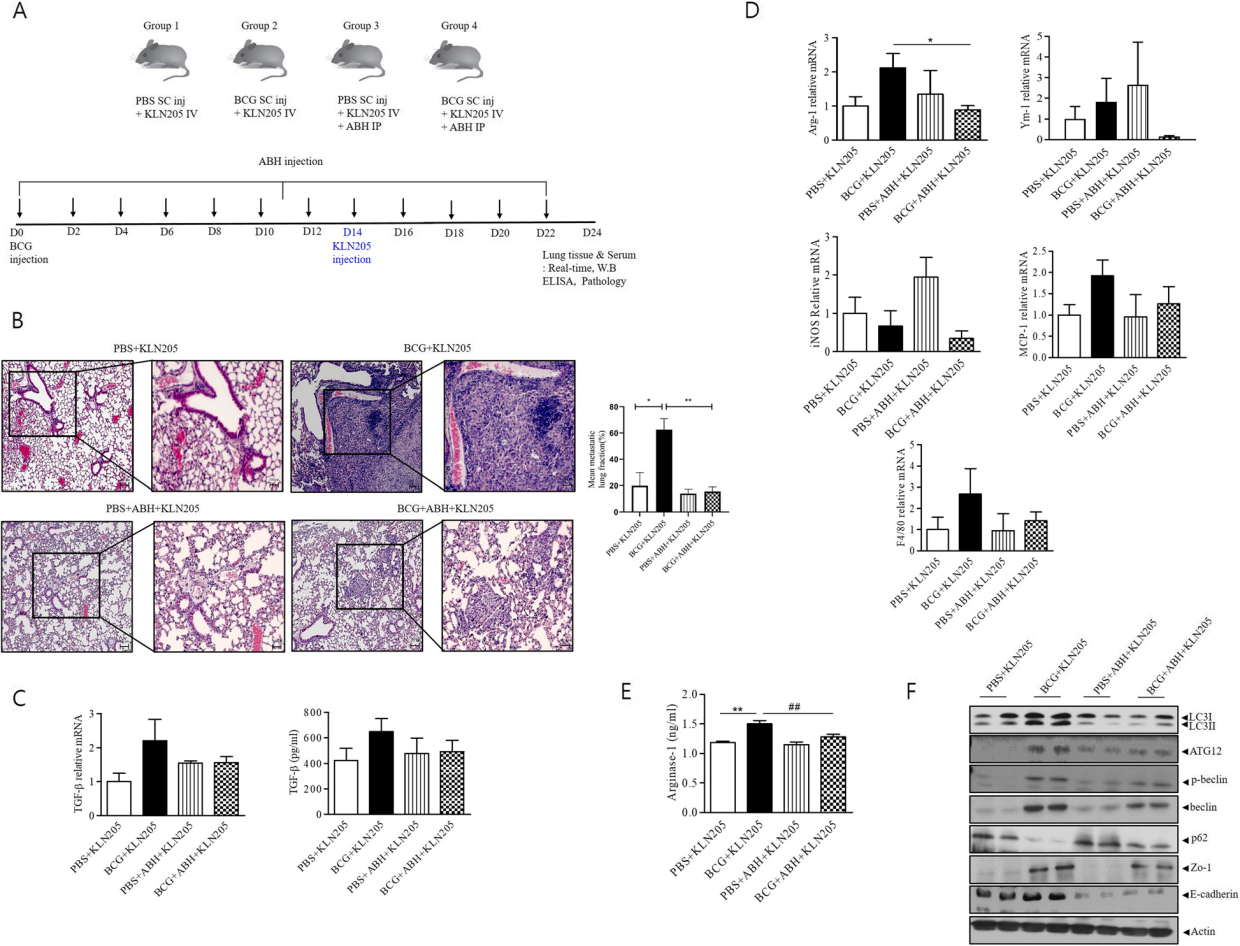
Arg-1 inhibition reduces BCG pleurisy-induced metastatic potential of lung cancer in vivo. **a** C57BL/6 mice were administered an intrapleural injection of PBS or BCG (1 × 10^6^ CFUs) following an i.p. injection of PBS or ABH 200 μg every 2 days for a total of 12 times and an intravenous injection of KLN205 mouse lung cancer cells (2 × 10^5^) on day 14. **b** Histological images of lungs. Hematoxylin and eosin staining (50 × , 200 ×). (Scale bar: left: 200um, right: 100um) Metastatic lung lesion was measured by calculating the total area of lung metastasis lesions by the total lung area. Statistically significant differences comparing four groups were analyzed using an unpaired Student’s t-test. * < 0.05, ** < 0.01 Data are shown as the mean ± standard deviation. **c** TGF-b mRNA expression and TGF-β ELISA. **d** The M2 (Arg-1 and YM-1), M1 (iNOS and MCP-1), and pan-macrophage (F4/80) markers were quantified in lung samples using RT-qPCR. **e** Western blotting of autophagy-related proteins (LC3II/LC3I, ATG12, p-Beclin, Beclin, and P62), ZO-1, and E-cadherin in lung cancer tissue lysates of each mouse (*n* = 7 per group). **p < 0.01, ##*p* < 0.01, *p < 0.05

The effects of Arg-1 inhibition on BCG pleurisy-associated lung cancer cells were evaluated in vivo. We treated KLN205 tumor-bearing C57BL6 mice after BCG injection with a commercially available Arg-1 inhibitor (ABH) at a dose of 200 μg i.p. every 2 days for a total of 12 times, and tumor growth rate was monitored over 22 days. Hematoxylin and eosin staining showed that ABH treatment with BCG injection resulted in a significant reduction in the number and size of lung metastatic nodules compared to BCG injection alone (Fig. 5a).

In accordance with the in vitro experimental results, the expression of Arg-1 mRNA expression was upregulated in the BCG + KLN205 group and downregulated in the BCG + ABH + KLN205 group (Fig. 5b). Among the MФ markers, the M2 marker Ym-1 and M1 marker iNOS did not show such a pattern, and MCP-1 and F4/80 levels tended to be lower in the BCG + ABH + KLN205 group than in the BCG + KLN205 group; however, the results were not significant. Similarly, the plasma arginase-1 level in the BCG + KLN205 group was higher than that in the PBS + _KLN205 group and its level in the BCG + ABH + KLN205 group was lower than that in the BCG + KLN205 group (Fig. 5c). The expression of the proinflammatory cytokine TGF-β, related to the progression of cancer, was reduced in the BCG + ABH + KLN205 group compared with that in the BCG + KLN205 group (Fig. 5d).

**Fig. 6:**
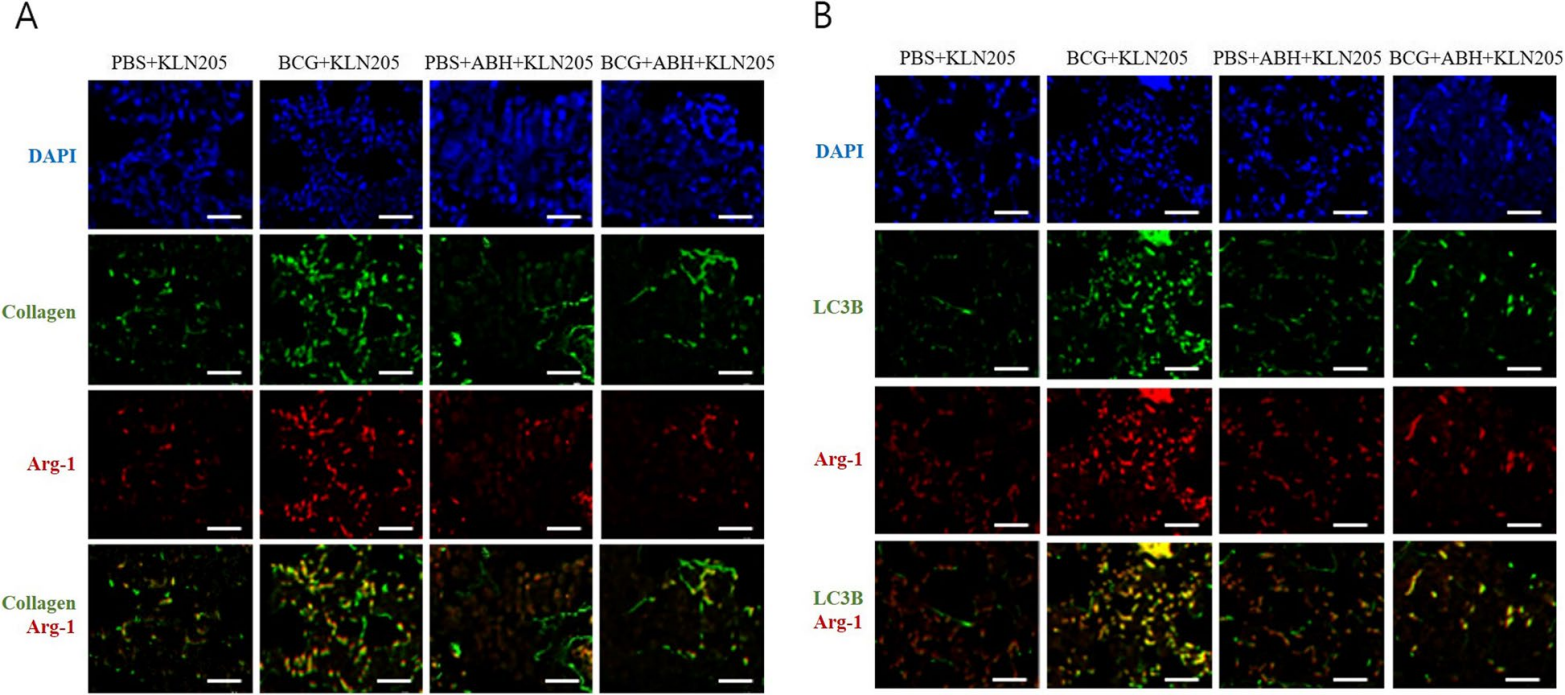
Activation of collagen and LC3 deposition is regulated by Arg-1 in a lung cancer model. **a** Immunofluorescence staining of collagen (green); DAPI staining (blue); Arg-1 staining (red). Yellow represents colocalization of Arg-1 and collagen. **b** Immunofluorescence staining of LC3B (green); DAPI staining (blue); Arg-1 staining (red). Yellow represents colocalization of Arg-1 and LC3B (Scale bar: 20um)

Arg-1 M2 polarization–autophagy/E-cadherin signaling is involved in TB-related lung cancer progression. Compared to the PBS + KLN205 group, the BCG + KLN205 group mice showed increased expression of ATG12-5, LC3II/LC3I, Beclin, E-cadherin, and ZO-1, but decreased expression of P62. In the BCG + ABH + KLN205 group, the expression of ATG12-5, LC3II/LC3I, Beclin, E-cadherin and ZO-1 decreased, but that of P62 increased, compared with those in the BCG + KLN205 group (Fig. 5f). Confocal immunofluorescence microscopy was used to confirm these findings. The lung sections of the BCG + KLN205 group had high levels of Arg-1 that colocalized with LC3B (Fig. 6). The inhibition of Arg-1 by ABH treatment attenuated LC3B expression induced by BCG + KLN205. In addition, the distribution of BCG-induced Arg-1 was similar to that of collagen. This finding suggests that Arg-1 MФ are associated with collagen production.

**Fig. 7:**
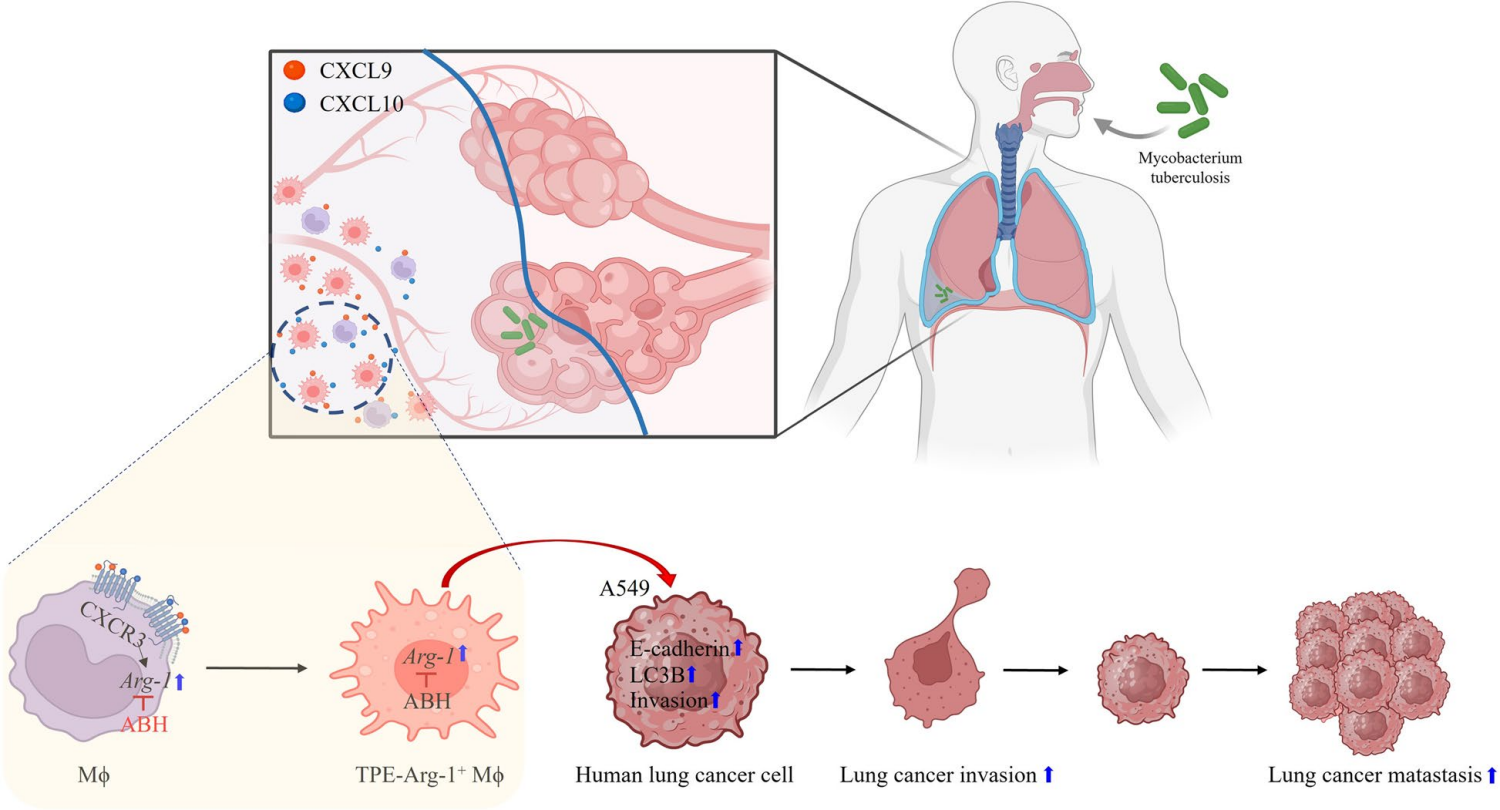
Schema of mechanism by which TPE-induced Arg-1^+^ macrophage polarization contributes to lung cancer malignancy. CXCL9 and CXCL10 are involved in TPE-induced macrophage M2 Arg-1 + polarization. TPE-Arg-1^+^ MФ CM induces A549 proliferation by upregulating autophagy and E-cadherin signaling. ABH reversed the effect of TPE-Arg-1^+^ MФ CM leading to cancer growth suppression by downregulating autophagy and E-cadherin signaling. ABH: 2(S)-amino-6-boronohexanoic acid (an arginase inhibitor)

In summary, we demonstrated that TPE induces Arg-1 M2 polarization of MФ via CXCL9/CXCL10, which promotes the progression of lung cancer via autophagy/E-cadherin signaling (Fig. 7).

## Discussion

The World Health Organization has reported that at least 20% of cancers have an infectious origin [14]. Several epidemiological studies have reported an association between TB and cancer development [2, 3, 15]. TB and cancer impose high socio-economic burden and are associated with high mortality rates [16]. Both diseases have been extensively researched; however, the overlap between them has not been examined in detail. An experimental study showed that chronic TB in mice could cause lung carcinogenesis by inducing DNA damage and epiregulin expression [17]. It has been hypothesized that TB causes lung cancer through genomic injury, cell death evasion, immune system suppression, and oncogene overexpression [16, 18]. However, robust experimental data are needed to establish a proof of concept.

In our previous study, we found that the NOX4–autophagy axis regulated by tuberculous fibrosis could result in enhanced tumorigenic potential [10, 16]. We hypothesized that after TB infection, the tumor microenvironment changes and becomes susceptible to tumor progression-inducing factors. Among the diverse immune cells that constitute the tumor microenvironment, MФ are particularly abundant and play a role in all stages of tumor progression. TAM can function as either tumoricidal agents (M1) or tumor-promoting immunosuppressants (M2) [19, 20]. TAMs provide a suitable microenvironment to induce growth, immunosuppression, invasion and chemotherapy resistance in lung cancer [21]. TAM secretes pro-angiogenic and tumor-inducing chemokines such as TGF-β, IL‑10, CCL18, matrix metalloproteases, epidermal growth factors and TGF- β [21, 22]. Also, TAM impede the immunoregulatory functions of The CD8+ T cells and tumor infiltrating lung dendritic cells [22–24]. Previous studies demonstrated the association between TAMs and chemotherapy resistance [25, 26]. Preclinical experiments have shown that targeting TAMs can be beneficial, as it can inhibit regulatory T-cell mechanisms and enhance the effect of anticancer therapy [9, 27–29].

During TB development, MФ exhibit phenotypic and functional heterogeneity depending on the site and stage of infection [30]. Robust innate immune responses in lung-resident MФ, monocytes, and monocyte-derived MФ play protective roles during TB development [30]. Meanwhile, based on the suppressive or hyperactivated immune status, the MTB virulence factor can affect MФ homeostasis and facilitate the development of granulomatous disease and spread of bacilli [31]. MTB promotes the M1-M2 switch of MФ to transform from an acute to chronic infection and develop drug resistance [32, 33]. Especially arginine metabolism plays a role in the differential regulation of MФ polarization [34]. Arginase, a pivotal metalloenzyme, metabolizes L-arginine to L-ornithine [35]. Thus, arginase plays an important role in fundamental cellular functions, such as wound healing and tissue fibrosis, and suppresses T-cell activation by locally depleting l-arginine [19].

MTB increased Arg-1 expression and substantially reduced bactericidal NO production by competing for l-arginine with i-NOS in a murine model [36]. Consistently, Pessanha et al. demonstrated the expression of Arg-1 in the granuloma of tissues samples obtained from patients with TB [37]. However, the exact role of Arg-1 in MTB-infected human lungs remains unknown. Some studies have reported the usefulness of L-Arg supplementation in patients with active TB; however, further research on its therapeutic and prophylactic roles is needed [38, 39].

In this study, MФ stimulation using tuberculous pleural fluid significantly increased Arg-1 MФ polarization, and similar results were observed in vivo. The cytokine assays and inhibition experiments confirmed that CXCL9 and CXCL10 present in TPE affected MФ Arg-1 polarization. A recent study also demonstrated that the CXCL9-CXCR3 axis plays a role in the migration and activation of MФ in an apical periodontitis model [40]. The CXCL9 and CXCL10/CXCR3 axes play two roles in the tumor environment: paracrine signaling for immune activation and autocrine signaling for cancer proliferation and metastasis [41]. Similar to a double-edged sword, CXCL9 not only inhibits tumor growth by recruiting CTL, but also contributes to cancer proliferation by recruiting Tregs, TAMs, and MDSCs, which are involved in immune tolerance in tumors [42].

Most importantly, we found that Arg-1^+^ MФ polarization induced by TPE contributed to lung cancer proliferation by enhancing autophagy signaling and E-cadherin expression. Arg-1^+^ MФ CM-treated A549 cells showed increased expression of LC3II/LC3I, ATG12-ATG5, Beclin 1, and E-cadherin, and reduced expression of P62. Autophagy has dual effects [43]. While cell intrinsic autophagy plays a role in preventing cancer proliferation at the initial stage, under stress conditions, autophagy is associated with tumorigenesis, tumor-stromal interactions, and chemoresistance in the tumor microenvironment [43]. Several studies have attempted to improve the anticancer effects of drugs by targeting specific autophagic processes [44, 45].

The downregulation of E-cadherin-mediated cell adhesion is related to epithelial–mesenchymal transition in tumor invasiveness [46]. On the other hands, E-cadherin plays a role in cancer progression via the PI3K-AKR and MEK-ERK pathways [46]. Padmanaban et al. showed that E-cadherin functions as a survival factor in breast cancer by reducing reactive oxygen species-mediated apoptotic signaling [47]. Similarly, the previous study demonstrated that high E-cadherin expression in the lung tumor was associated with worse overall survival in patients with stage IV EGFR-mutant lung adenocarcinoma [48]. The complicated link between autophagy and EMT exists [49]. Autophagy activation can suppress or promotes EMT by regulating various signaling pathways. However, in this study, the increase in E-cadherin in tuberculous fibrosis-induced lung cancer appears to be related to cell basal extrusion, local invasion, distant metastasis, and tumor cell circulation rather than a complete EMT process [50]. High levels of autophagy in oncogenic K-ras cells promotes basal extrusion of epithelial cells that is related to tumor promoting functions of E-cadherin [51].

Treatment with an Arg-1 inhibitor reduced the expression of LC3II/LC3I, ATG12, Beclin 1, and E-cadherin and increased the expression of p62 in this study. We found that the Arg-1 inhibitor suppressed cancer progression by modulating autophagy and E-cadherin signaling in TB-associated lung cancer. Based on these findings, TB-associated lung cancer appears to have anticancer drug-resistance properties and specific molecular characteristics.

Previous studies have reported that immunotherapy resistance can be overcome by modulating myeloid cells and TAM [20, 52]. Further studies on TB-associated lung cancer are needed to confirm chemoresistance and improvement of treatment efficacy when an Arg-I inhibitor is used in combination with other drugs such as immune checkpoint inhibitors.

## Conclusions

We found that Arg-1 MФ play an important role in the progression of lung cancer in TB-associated microenvironments. TB-associated lung cancer is associated with autophagy and E-cadherin signaling, which are related to chemoresistance. Our results showed the translational potential of Arg-1 inhibitors for TB-associated lung cancer and demonstrated a challenge for the metabolic regulation of immune responses in both TB and lung cancer. Further research is required to evaluate the chemoresistance and stemness of TB-associated lung cancer, which will ultimately translate into enhanced therapeutics.

### Supplementary Information

Supplementary Material 1: Supplementary Table 1. Mouse or human sequences and accession numbers for primers (forward, FOR; reverse, REV) used in real-time RT-PCR.

Supplementary Material 2: Supplementary Figure 1. Changes in macrophage polarization by day after pleural effusion treatment on BMDM. (a) Cell proliferation rate of macrophages after TPE or T (Transudate) treatment from Day 0 to Day 3. (b) The specific M2 (Arg-1 and YM-1) markers, M1 (iNOS) markers and pan-macrophage markers (CD68) were quantified by RT-qPCR after stimulation with TPE or T. The values represent the results of three experiments. TPE vs T: **p < 0.01, *p < 0.05, D0 vs other time: #*p* < 0.05.

Supplementary Material 3: Supplementary Figure 2. Effect of 3-methyladenine (3-MA) and bafilomycin (BafA1) on autophagy in TPE-Arg-1+ MФ CM-treated A549 cells. A549 cells were treated with vehicle control or 10 nM BafA1 or 10 mM 3-Ma in the presence or absence of TPE-Arg-1+ MФ CM for 48 h. Thereafter, protein expression was examined using western blot analysis of LC3II /LC3I, ATG-12, and actin.

Supplementary Material 4: Supplementary Figure 3. Interaction between macrophage and A549 cell after TPE treatment. Transwell assay showed that A549 cells promote migration of TPE-Arg-1 + MФ compared to T-Arg-1 – MФ. TPE-Arg-1++ MФ or T-Arg-1-+ MФ cells were seeded (1 × 10^5^ cells/well) in the upper chamber and the A549 cells were seeded to the lower chamber.

Supplementary Material 5.

## Data Availability

The data underlying this article will be shared on reasonable request to the corresponding author.

## Abbreviations

3-MA: 3-Methyladenine
ABH: Amino-6-boronohexanoic acid
BafA1: Bafilomycin A1
BCG: Bacillus Calmette–Guérin
BMDM: Bone marrow-derived macrophage
CM: Culture medium
MФ: Macrophage
PRC: Polymerase chain reaction
TAM: Tumor-associated macrophages
TB: Tuberculosis
TPE: Tuberculous pleural effusion
