# Supplementary material for: Tuberculous pleural effusion-induced Arg-1+ macrophage polarization contributes to lung cancer progression via autophagy signaling
Source: Respir Res. 2024 May 8;25:198. doi: 10.1186/s12931-024-02829-8 (PMC11077851; doi:10.1186/s12931-024-02829-8)
Supplement: Supplementary file 1 — Supplementary Material 1: Supplementary Table 1. Mouse or human sequences and accession numbers for primers (forward, FOR; reverse, REV) used in real-time RT-PCR. [file 12931_2024_2829_MOESM1_ESM.docx]

**Supplementary table 1. Sequences and accession numbers for primers (forward, FOR; reverse, REV) used in real-time RT-PCR**

| Gene | Primer sequences (5′-3′) |
| --- | --- |
| *Mu.Arg-1* | FOR: CAGAAGAATGGAAGAGTCAG |
|  | REV: CAGATATGCAGGGAGTCACC |
| *Mu.Ym-1* | FOR: GGGCATACCTTTATCCTGAG |
|  | REV: CCACTGAAGTCATCCATGTC |
| *Mu.Nos2* | FOR: TTCTGTGCTGTCCCAGTGAG |
|  | REV: TGAAGAAAACCCCTTGTGCT |
| *Mu.Ccl2* | FOR: ATTGGGATCATCTTGCTGGT |
|  | REV: CCTGCTGTTCACAGTTGCC |
| *Mu.CD68* | FOR: CAAAGCTTCTGCTGTGGAAAT |
|  | REV: GACTGGTCACGGTTGCAAG |
| *Mu.Adgre1* | FOR: TTTCCTCGCCTGCTTCTTC |
|  | REV: CCCCGTCTCTGTATTCAACC |
| *Mu.CXCR3* | FOR: CAGCCTGAACTTTGACAGAACCT |
|  | REV: GCAGCCCCAGCAAGAAGA |
| *Mu.TGFβ* | FOR: GGATACCAACTATTGCTTCAGCTCC |
|  | REV: AGGCTCCAAATATAGGGGCAGGGTC |
| *Mu.GAPDH* | FOR: CGTCCCGTAGACAAAATGGT |
|  | REV: TTGATGGCAACAATCTCCAC |
| *Hu.Arg-1* | FOR: GTTTCTCAAGCAGACCAGCC |
|  | REV: GCTCAAGTGCAGCAAAGAGA |
| *Hu.Actin* | FOR: GTGCTATCCCTGTACGCCTC |
|  | REV: GGCCATCTCTTGCTCGAAGT |
