# Supplementary material for: Tuberculous pleural effusion-induced Arg-1+ macrophage polarization contributes to lung cancer progression via autophagy signaling
Source: Respir Res. 2024 May 8;25:198. doi: 10.1186/s12931-024-02829-8 (PMC11077851; doi:10.1186/s12931-024-02829-8)
Supplement: Supplementary file 2 — Supplementary Material 2: Supplementary Figure 1. Changes in macrophage polarization by day after pleural effusion treatment on BMDM. (a) Cell proliferation rate of macrophages after TPE or T (Transudate) treatment from Day 0 to Day 3. (b) The specific M2 (Arg-1 and YM-1) markers, M1 (iNOS) markers and pan-macrophage markers (CD68) were quantified by RT-qPCR after stimulation with TPE or T. The values represent the results of three experiments. TPE vs T: **p < 0.01, *p < 0.05, D0 vs other time: #p < 0.05. [file 12931_2024_2829_MOESM2_ESM.docx]

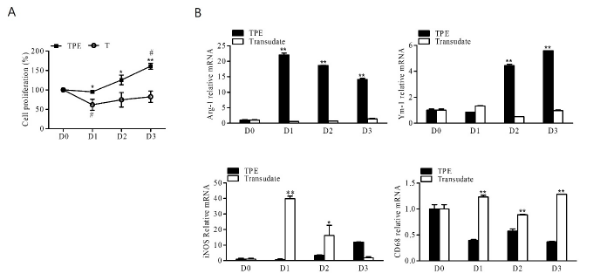


**Supplementary Figure 1.  Changes in macrophage polarization by day after pleural effusion treatment on BMDM**

(a) Cell proliferation rate of macrophages after TPE or T (Transudate) treatment from Day 0

to Day 3. (b) The specific M2 (Arg-1 and YM-1) markers, M1 (iNOS) markers and pan macrophage markers (CD68) were quantified by RT-qPCR after stimulation with TPE or T. The values represent the results of three experiments. TPE vs T: **p< 0.01, *p<0.05, D0 vs other time: #p< 0.05
