# Supplementary material for: Tuberculous pleural effusion-induced Arg-1+ macrophage polarization contributes to lung cancer progression via autophagy signaling
Source: Respir Res. 2024 May 8;25:198. doi: 10.1186/s12931-024-02829-8 (PMC11077851; doi:10.1186/s12931-024-02829-8)
Supplement: Supplementary file 3 — Supplementary Material 3: Supplementary Figure 2. Effect of 3-methyladenine (3-MA) and bafilomycin (BafA1) on autophagy in TPE-Arg-1+ MФ CM-treated A549 cells. A549 cells were treated with vehicle control or 10 nM BafA1 or 10 mM 3-Ma in the presence or absence of TPE-Arg-1+ MФ CM for 48 h. Thereafter, protein expression was examined using western blot analysis of LC3II /LC3I, ATG-12, and actin. [file 12931_2024_2829_MOESM3_ESM.docx]

**
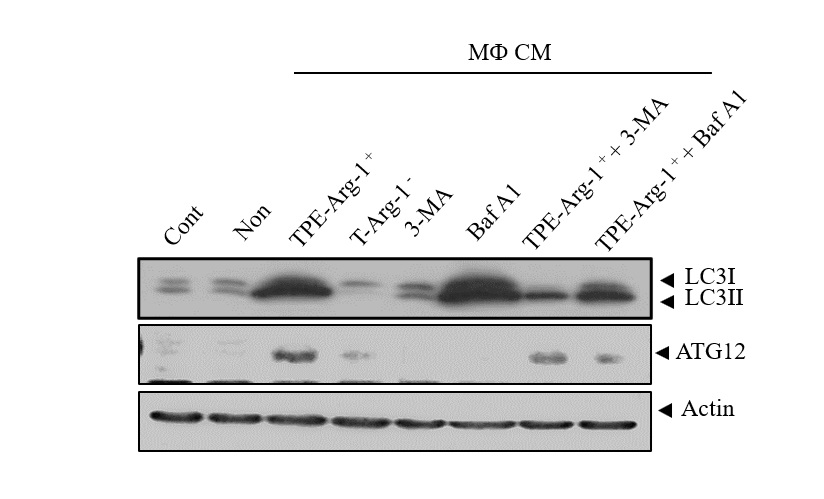
**

**Supplementary Figure 2. Effect of 3-methyladenine(3-MA) and bafilomycin(BafA1) on autophagy in TPE-Arg-1 ^+^ MФ CM treated A549 cells**

A549 cells were treated with vehicle control or 10 nM BafA1 or 10mM 3-Ma in the presence or absence of TPE-Arg-1 ^+^ MФ CM for 48 hours. Thereafter, protein expression was examined using western blot analysis for LC3II /LC3I, ATG-12 and actin.
