# Supplementary material for: Tuberculous pleural effusion-induced Arg-1+ macrophage polarization contributes to lung cancer progression via autophagy signaling
Source: Respir Res. 2024 May 8;25:198. doi: 10.1186/s12931-024-02829-8 (PMC11077851; doi:10.1186/s12931-024-02829-8)
Supplement: Supplementary file 4 — Supplementary Material 4: Supplementary Figure 3. Interaction between macrophage and A549 cell after TPE treatment. Transwell assay showed that A549 cells promote migration of TPE-Arg-1 + MФ compared to T-Arg-1 – MФ. TPE-Arg-1++ MФ or T-Arg-1-+ MФ cells were seeded (1 × 10^5^ cells/well) in the upper chamber and the A549 cells were seeded to the lower chamber. [file 12931_2024_2829_MOESM4_ESM.docx]

**
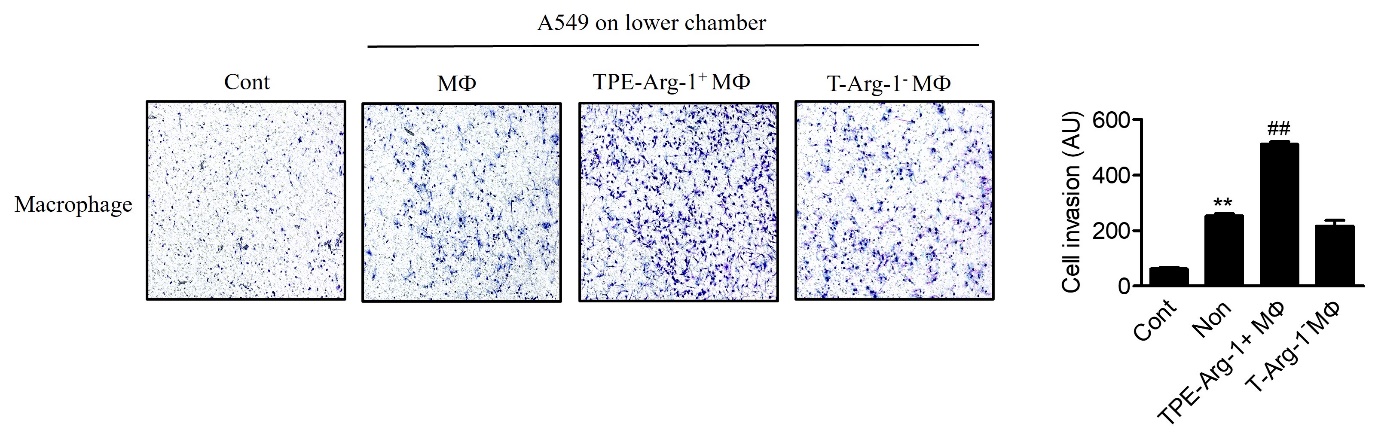
ASupplementary Figure 3. Interaction between macrophage and A549 cell after TPE treatment**

Transwell assay showed that A549 cells promote migration of TPE-Arg-1 ^+^ MФ compared to T-Arg-1 ^–^ MФ. TPE-Arg-1+ MФ or T-Arg-1-+ MФ cells were seeded (1×10^5^ cells/well) in the upper chamber and the A549 cells were seeded to the lower chamber.
