# Supplementary figures and images for: Tuberculous pleural effusion-induced Arg-1+ macrophage polarization contributes to lung cancer progression via autophagy signaling
Source: Respir Res. 2024 May 8;25:198. doi: 10.1186/s12931-024-02829-8 (PMC11077851; doi:10.1186/s12931-024-02829-8)

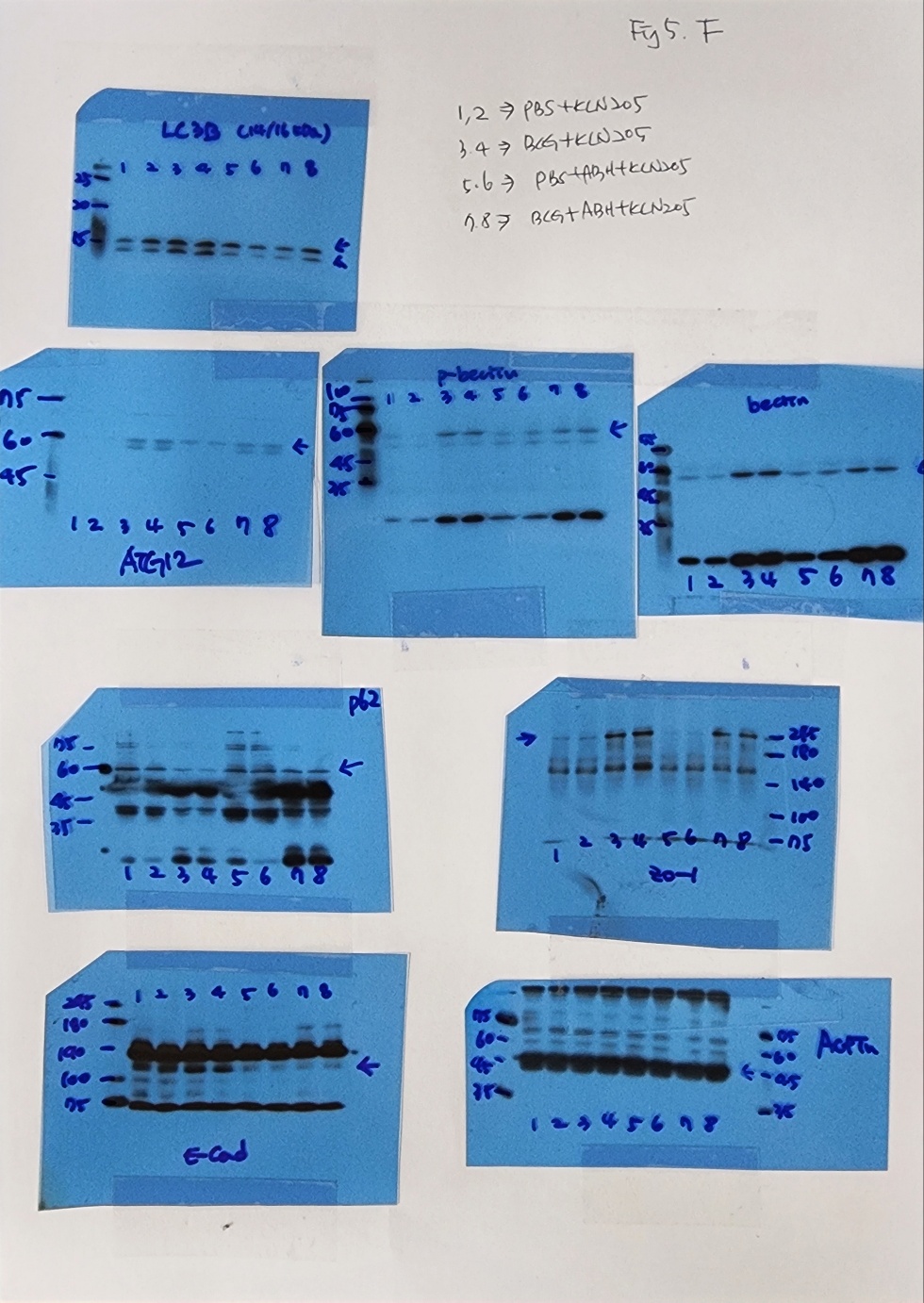

Supplement: Supplementary file 5 — Supplementary Material 5. [file 12931_2024_2829_MOESM5_ESM.zip › complete Fig5 WB.jpg]

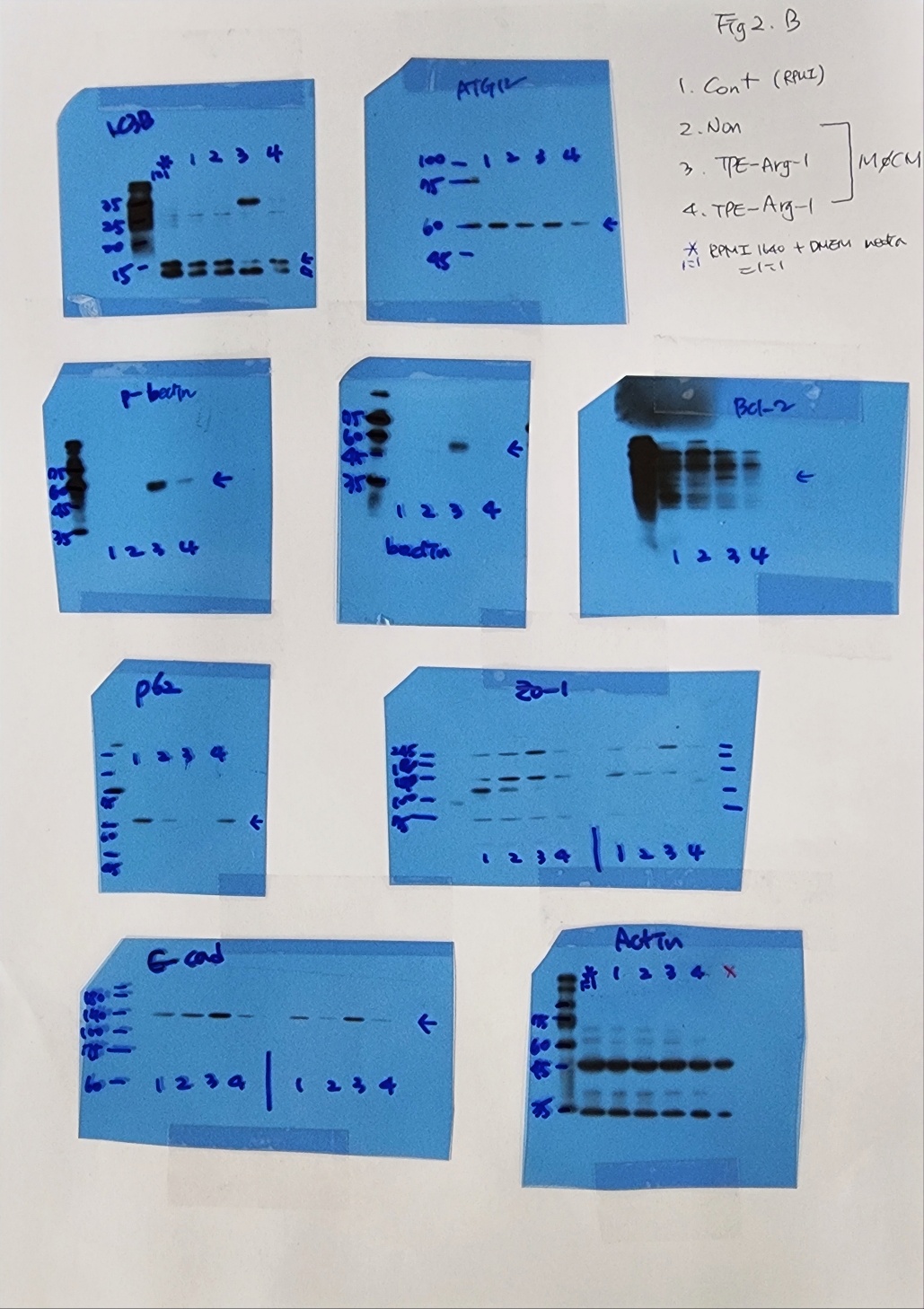

Supplement: Supplementary file 5 — Supplementary Material 5. [file 12931_2024_2829_MOESM5_ESM.zip › Fig2b WB.jpg]

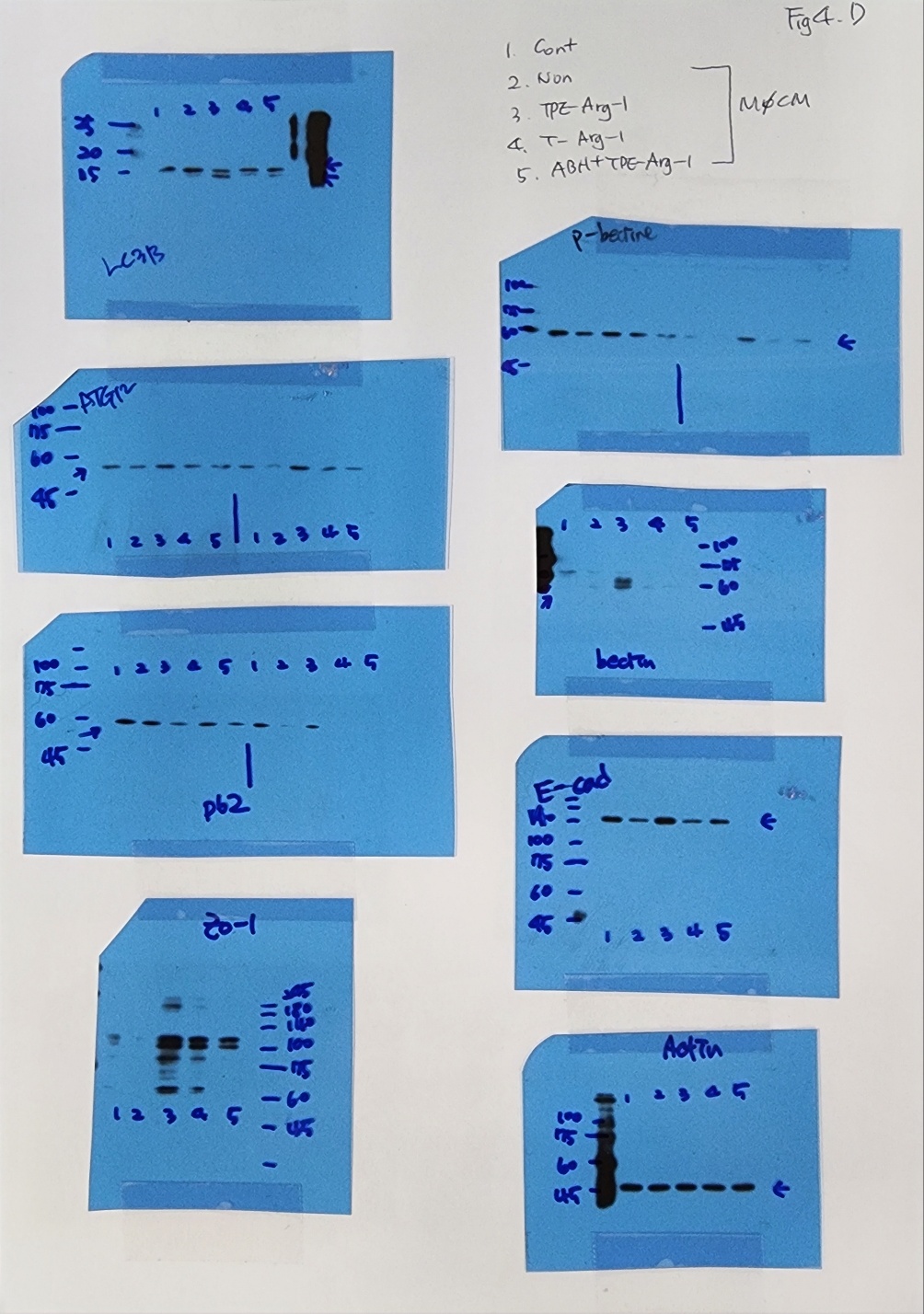

Supplement: Supplementary file 5 — Supplementary Material 5. [file 12931_2024_2829_MOESM5_ESM.zip › Fig4d WB.jpg]

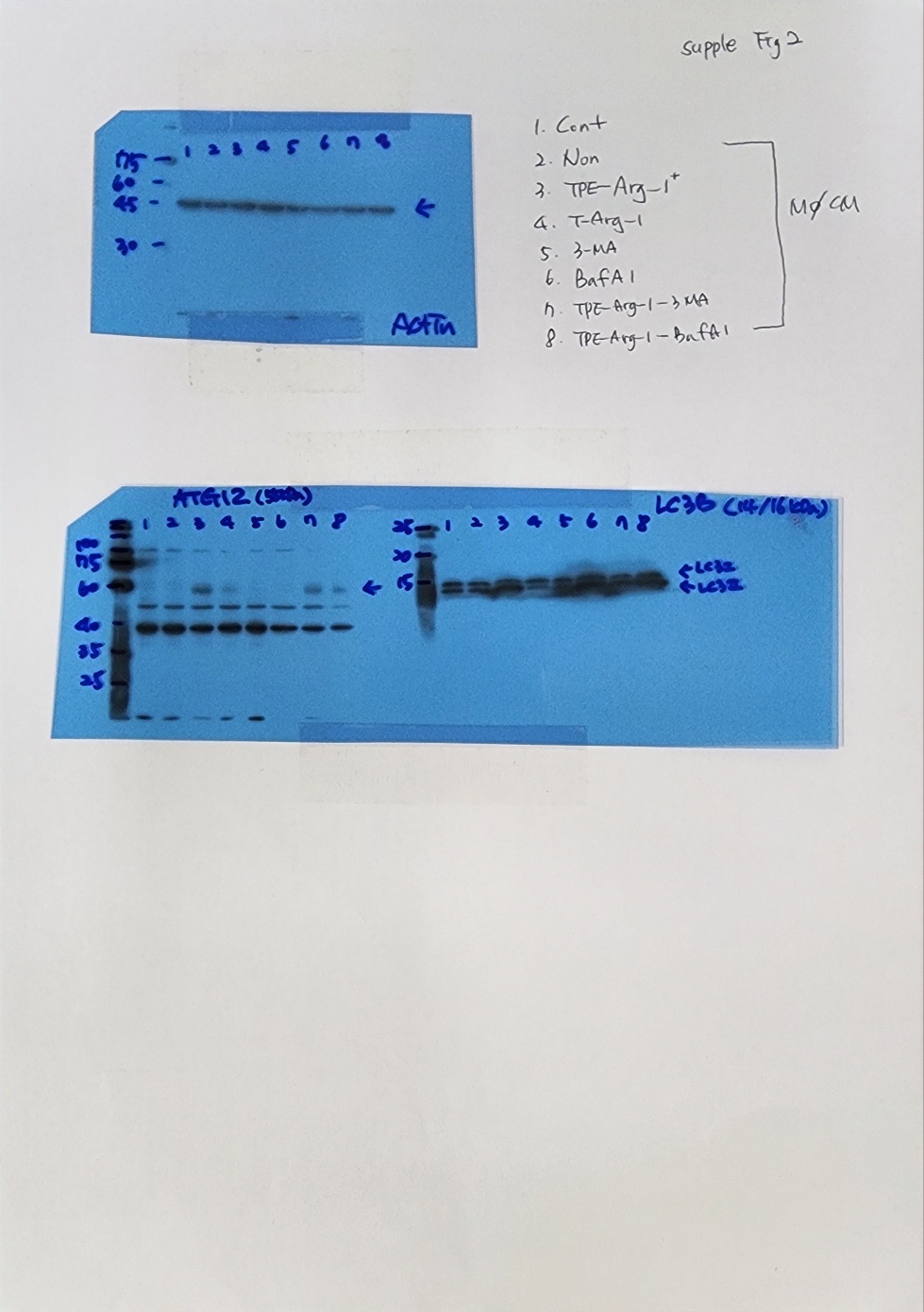

Supplement: Supplementary file 5 — Supplementary Material 5. [file 12931_2024_2829_MOESM5_ESM.zip › Supplementary Figure 2 WB.jpg]
